# Effects of hyperthermic baths on depression, sleep and heart rate variability in patients with depressive disorder: a randomized clinical pilot trial

**DOI:** 10.1186/s12906-017-1676-5

**Published:** 2017-03-28

**Authors:** Johannes Naumann, Julian Grebe, Sonja Kaifel, Tomas Weinert, Catharina Sadaghiani, Roman Huber

**Affiliations:** 1 Interdisciplinary Center for Treatment and Research in Balneology, Institute for Environmental Health Sciences and Hospital Infection Control, Medical Faculty, Medical Center University of Freiburg, Breisacher Straße 115b, Freiburg im Breisgau, 79106 Germany; 2 Center for Complementary Medicine, Institute for Environmental Health Sciences and Hospital Infection Control, Medical Faculty, Medical Center University of Freiburg, Breisacher Straße 115b, Freiburg im Breisgau, 79106 Germany

**Keywords:** Depression, Hyperthermic baths, Heart rate variability, RCT

## Abstract

**Background:**

Despite advances in the treatment of depression, one-third of depressed patients fail to respond to conventional antidepressant medication*.* There is a need for more effective treatments with fewer side effects. The primary aim of this study was to determine whether hyperthermic baths reduce depressive symptoms in adults with depressive disorder.

**Methods:**

Randomized, two-arm placebo-controlled, 8-week pilot trial. Medically stable outpatients with confirmed depressive disorder (ICD-10: F32/F33) who were moderately depressed as determined by the 17-item Hamilton Scale for Depression (HAM-D) score ≥18 were randomly assigned to 2 hyperthermic baths (40 °C) per week for 4 weeks or a sham intervention with green light and follow-up after 4 weeks. Main outcome measure was the change in HAM-D_total score_ from baseline (T0) to the 2-week time point (T1).

**Results:**

A total of 36 patients were randomized (hyperthermic baths, *n* = 17; sham condition, *n* = 19). The intention-to-treat analysis showed a significant (*P* = .037) difference in the change in HAM-D_total score_ with 3.14 points after 4 interventions (T1) in favour of the hyperthermic bath group compared to the placebo group.

**Conclusions:**

This pilot study suggests that hyperthermic baths do have generalized efficacy in depressed patients.

**Trial registration:**

DRKS00004803at drks-neu.uniklinik-freiburg.de, German Clinical Trials Register (registration date 2016-02-02), retrospectively registered.

**Electronic supplementary material:**

The online version of this article (doi:10.1186/s12906-017-1676-5) contains supplementary material, which is available to authorized users.

## Background

Depression is the leading cause of disability worldwide, and a major contributor to the global burden of disease, with more than 350 million people of all ages suffering from depression [1]. The associated economic burden is estimated at $317 billion annually [2], and antidepressant medications have become the most commonly prescribed treatment in medical practice [3]. In addition, psychiatric conditions, particularly depressive disorders, are associated with increased prevalence of chronic diseases and often precipitate or exacerbate these conditions [4]. Depression is a known risk factor for the development of cardiovascular disease (CVD) [5], and patients with CVD and depression have a two to threefold increased risk of future cardiac events compared to cardiac patients without depression [6].

Alterations in the autonomic nervous system have been hypothesized to be an underlying physiological mechanism that may partly explain these unfavorable health outcomes among depressed persons. Such alterations are believed to disturb circadian functioning, sleep and temperature physiology [7]. Depressed persons are found to have a reduced heart rate variability (HRV), a well-known prognostic risk factor for CVD and mortality; however, it is unclear whether this is a consequence of the disorder or due to antidepressant medication [8, 9]. Most depressed patients also report disturbances in their sleep, such as difficulties in falling asleep, waking during the night or early morning awakenings. Thus, sleep disturbance is an important mechanism contributing to depression [7, 10]. Furthermore, body core temperature in depressed patients is elevated during the night; thus, change of body temperature might influence depression [11, 12]. Despite advances in the treatment of depression, one-third of depressed patients fail to respond to conventional antidepressant medication [13].

Moreover, current treatments come at the cost of significant central nervous side effects, further highlighting the need for more effective treatments with fewer side effects [14].

As a conclusion, there is a need for additional (non-pharmacological) treatment of depressive patients that positively influence cardiovascular risk factors, HRV and quality of sleep. Evidence suggests that hyperthermic baths (HTB) and other forms of whole body hyperthermia (WBH) have antidepressant effects, mediated through changes in circadian functioning and temperature physiology, although the underlying mechanisms remain unclear [15, 16].

Results of a non-controlled HTB study with 20 depressive patients showed an improvement in the 21-item Hamilton Depression Scale [17] after five baths [18]. Furthermore, HTB (especially before bedtime) improved sleep in healthy subjects [19–22], insomniac people [23, 24] and elderly patients with vascular dementia [25]. In a further non-controlled study using a radiant system to induce WBH, a single session showed a significant reduction in the Centers for Epidemiologic Studies Depression Scale (CES-D) [26] in 16 depressive patients [27].

Results of these uncontrolled studies are promising; therefore, we conducted this randomized sham-placebo-controlled pilot study that included assessment of depressive symptoms, sleep quality and HRV parameters. Our hypotheses were 2-fold. First, we expected HTB to lower depressive symptoms. Second, we expected this to be mediated by improved sleep and circadian functioning, evoked by changes in HRV parameters.

## Methods

### Study design and participants

Because of the unknown effect size and in order to evaluate the feasibility of recruitment and assessment procedures a pilot study was conducted.

This pilot study was a single-site, parallel-group, randomized controlled trial of HTB vs sham-placebo (green light) for patients with a confirmed diagnosis of depression according to ICD-10 (F32/F33) of at least 4 weeks duration. Recruitment via public announcements took place from June 2013 until September 2013.

Eligible participants were men and women between the ages of 18 and 65 who had been on a consistent antidepressant regimen or had been off antidepressant therapy for at least 4 weeks prior to baseline. No changes in antidepressant treatment were allowed during the study. All participants were required to have a total score ≥ 18 and a score ≥ 2 on item 1 (Depressed Mood) at screening and at baseline, assessed by the 17-item Hamilton Scale for Depression (HAM-D). Exclusion criteria included the presence of severe concomitant disease, epileptic disorders, organic psychotic disorders, schizophrenia, hallucinations, bipolar disorders, dissociative personality disorder, suicidal thoughts, abuse of alcohol or other drugs within the last 6 months, use of ß-blockers or corticosteroids, open wounds, heat urticaria, pregnancy, lactation, aversion to hot baths and participation in clinical trials in the 8 weeks preceding the study.

### Ethics statement

Ethical approval was obtained from the local Ethics Committee (Ethics-Commission Medical Center University of Freiburg; 96/13; 22.04.2013). The study was retrospectively registered in the German Clinical Trials Register (DRKS) with the registration number DRKS00004803. The study was conducted in accordance with the Declaration of Helsinki and local laws and regulations. All participants gave written informed consent. The full study protocol can be found in Additional file 1.

### Interventions

Patients were randomly assigned to receive either HTB or green light therapy (sham condition) for 4 weeks with 2 interventions per week (8 interventions in each group). Follow-up took place 4 weeks after the last intervention (see Table 1). Patients were told that two promising treatments are compared in the study.

**Table 1:** Diagram of the study protocol

			2 weeks	2 weeks	4 weeks
	Screening	Baseline/Randomization	After 4 interventions		Follow-up
		T0	T1		T2
HTB target (n)^a^			4	4	
17-item HAM-D	x	x	x		x
Medical history^b^		x			
HRV^c^		x	x		x
Global judgement of efficacy			x		x
Global judgement of tolerability			x		x
Adverse events^d^			x	x	x

Assessments of psychometry and heart rate variability parameters (HRV) were conducted at baseline (T0), after 2 weeks of treatment (T1), and 4 weeks after discontinuation of treatment (T2). Note that assessments were not conducted after 4 weeks of treatment

^a^ HTB: hyperthermic baths; temperature of bath; ear-temperature before/after bath; ear-temperature after rest; duration of bath (target 30 min); duration of rest

^b^Duration of depression; approved diagnosis family/consulting physician; intake of psychometric drugs no/yes; psychotherapy no/yes; psychiatric hospital stays

^c^ Application of portable ECG for overnight measurement

^d^ Adverse events were documented before and after each treatment

#### Hyperthermic baths

The hyperthermic baths were applied as head-out-of-water-immersion in a 40 °C pool at a spa center near Freiburg, Germany. All the baths were conducted in the afternoon (14:00–18:00). Five patients were able to sit in the pool at a time. The baths were taken until the participants noticed discomfort, the target being 30 min. Directly after the bath, the participants were accompanied to a nearby resting room, where they lay down on a resting lounger and were wrapped in warm blankets with 2 conventional 0.7 l hot water bottles (abdomen, thighs) filled with boiling water for at least another 30 min to keep the body temperature elevated. After 20–30 min in a hyperthermic bath with a water temperature of 40 °C a raise in core body temperature of 1.7 °C can be expected [28].

The following parameters were controlled and documented: water temperature in the bath, duration of bath and resting time.

#### Core body temperature

Core body temperature was measured with an infrared-ear-thermometer (Thermoscan®, Type: 6021, Braun GmbH), before and after the bath and after resting.

#### Sham intervention

Participants received a sham green LED light intervention (<400 Lux; <40 min) in group settings in a sitting position at a university outpatient’s department.

Therapeutic effects can be expected for therapies at 10 000 Lux/30 min per day [29].

### Outcome measures

Unblinded assessments were performed at the following 3 time points (see Table 1): before the start of HTB treatment (T0), immediately on completion of the 2-week treatment interval (T1), according to results that effects are supposed to appear early, [15] and 4 weeks after the end of treatment (T2).

The primary outcome was determined to be the change in HAM-D_total score_ at T1 relative to T0. Secondary efficacy outcome measures were (1) change in HAM-D_total score_ at T2 relative to T0 to investigate whether immediate responses would last after treatment discontinuation, (2) change in HAM-D subscales (T1 and T2): Insomnia as a symptom was measured by a HAM-D subscale (range of scores 0–4) defined by the cluster of items 4 (Early in the Night), 5 (Middle of the Night), and 6 (Early Hours of the Morning). Low mood, a core symptom of depression, was assessed by a HAM-D subscale consisting of items 1 (Depressed Mood), 2 (Guilt), 3 (Suicide), 7 (Loss of Interest), and 10 (Anxiety). Somatic complaints were measured by a HAM-D subscale defined by the cluster of items 11 (Anxiety Somatic), 12 (Gastro-intestinal Symptoms), and 13 (General Symptoms) [30, 31].

#### Heart rate variability outcome measures

Further secondary endpoints were standard HRV parameters, notably total heart rate variability (SDNN; standard deviation of normal-to-normal R-R intervals), low frequency (LF), high frequency (HF), LF/HF ratio and heart rate (HR; mBPM) during sleep (T1 and T2: 21:00-04:00, 21:00-0:30 and 0:30-4:00). We chose these night-time intervals because we were interested in sleep patterns and because these measurements are supposed to show less artefacts and influence of daily life activity.

HRV was measured with a portable high-frequency recorder “Medilog® AR12plus” (Schiller Medizintechnik GmbH, Germany), with a resolution of 1000 Hz. Electrodes were fixed at 5 locations (upper and lower end of sternum, below lateral third of left clavicle, under ribs right and left of left mamillar line). HRV analysis was performed with the program “Medilog Darwin Version 1.13.4”.

#### Global judgement of efficacy and tolerability

After 4 treatments (T1) and 4 weeks after end of treatment (T2), participants were asked to rate the efficacy and tolerability of the intervention on a 5-point scale (1 indicates very good; 2, good; 3, moderate; 4, absent; 5, worsening).

#### Adverse events

Adverse events (AE) were documented before and after each treatment by two unblinded assistants.

### Statistical analyses

#### Sample size

We calculated the sample size assuming an effect of one standard deviation (SD) between the groups based on a two-sided *t* test with an α-risk level of .05 and a statistical power of 80%, requiring 17 participants per group. With an expected dropout rate of 20%, 22 participants per group were chosen. Because of slow recruitment and logistical difficulties, however, the study was halted before the target sample size was attained.

#### Randomization and blinding

Randomization codes were computer-generated by an independent biometric center. Allocation was performed with opaque sealed envelopes that were randomly chosen by the participants. Both therapies could not be blinded. Data management and analyses were performed blinded to treatment allocation.

#### Data analyses

Efficacy parameters were analyzed based on the intention-to-treat (ITT) population, defined as all allocated participants, applying the last-observation-carried-forward approach to impute missing data. Baseline characteristics were compared using 2-sided *t* tests for continuous data and χ^2^ statistics. The per-protocol (PP) population was defined as all participants who had a complete dataset for the relevant parameters and had participated in at least 75% of the treatments, meaning at least 3 of 4 treatments for T1, and at least 6 of 8 treatments for T2.

Based on the fact that the HTB group was expected to show better performance than the placebo group, we justified 1-sided testing on the primary and secondary outcomes of depression ratings. We report *P*-values with the significance level set at *P* < .05 and Cohen *d* [32] as effect size. Correlation analyses were used to explore associations between HAM-D scores and HRV parameters. Secondary analyses were not adjusted for multiple comparisons and should therefore be regarded as descriptive and exploratory. Where not otherwise indicated, data are expressed as mean (SD). Statistical analyses were performed using SPSS®, Version 22, for WindowsTM.

## Results

### Study population

**Fig. 1:**
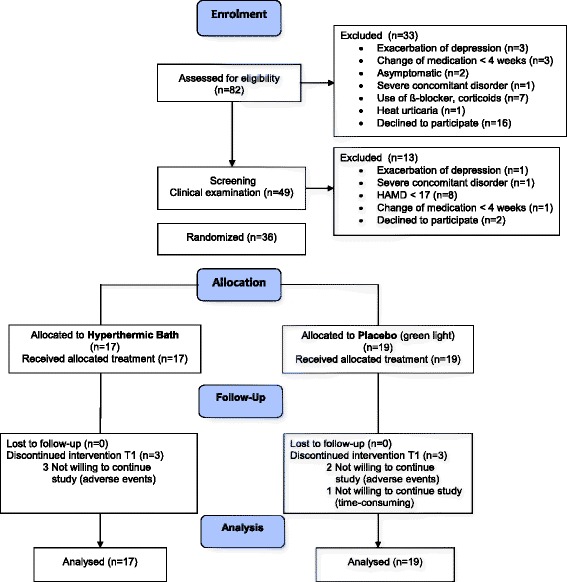
CONSORT Flow Diagram of study participants

We included and randomized 36 depressed patients (HTB, *n* = 17; placebo, *n* = 19). Six patients (HTB, *n* = 3; placebo, *n* = 3) discontinued treatment before T1; none were lost to follow-up (see Fig. 1). Randomization was balanced with respect to demographic and clinical characteristics (Table 2).

**Table 2:** Baseline demographic and clinical characteristics of the 36 randomized study patients (intention-to-treat population) by treatment assignment

	Overall *N* = 36	HTB Group (*N* = 17)	Placebo Group (*N* = 19)	*P* Value^a^
Gender (female/male), n	28/8	14/3	14/5	0.70
Age (years)	47 (11.9)	44 (13.0)	49 (10.6)	0.22
BMI (kg/m^2^)	24 (5.2)	24 (3.9)	25 (6.3)	0.72
Duration of depression (months)	93 (85.7)	98 (92.5)	89 (82.7)	0.79
Use of psychopharmaca (%)	64	65	63	1
HAM-D_total score_	22.36 (3.7)	23.06 (3.9)	21.74 (3.4)	0.29
HAM-D_insomnia_	3.36 (2.0)	3.35 (1.8)	3.37 (2.3)	0.98
HAM-D_mood_	10.47 (2.1)	10.82 (1.8)	10.16 (2.3)	0.35
HAM-D_somatic_	3.92 (1.2)	4.00 (1.3)	3.84 (1.2)	0.71
Heart rate (21:00-04:00)^b^	72.77 (10.8)	73.20 (9.4)	72.39 (12.2)	0.84
HRV LF (21:﻿00﻿-04:00)^b^	490.30 (329.8)	485.70 (353.6)	494.33 (319.3)	0.94
HRV HF (21:﻿00﻿-04:00)^b^	161.94 (133.8)	156.47 (154.6)	166.72 (117.6)	0.84
HRV SDNN (21:﻿00﻿-04:00)^b^	55.14 (16.5)	55.33 (15.9)	54.97 (17.6)	0.95

Where not otherwise indicated, data are shown as mean and standard deviation (SD)

*Abbreviations: HTB* hyperthermic baths, *BMI* body mass index (calculated as weight in kilograms divided by height in meters squared)

^a^Calculated as comparisons of HTB and placebo, using 2-tailed *t* tests (continuous variables) or χ^2^ tests (discrete variables)

^b^Night-time intervals

### Treatment effect on core body temperature

Core body temperature rose from 36.6 °C before the baths to 39.1 °C directly after the baths (mean change 2.43 [0.4]) and maintained at 37.7 °C (mean change 1.06 [0.5]) after rest. The mean temperature of the bath was 40.2 °C (0.3). Mean duration of baths was 22.6 (3.5) min and resting time amounted to 33.2 (6.3) min.

### Primary outcome

**Fig. 2:**
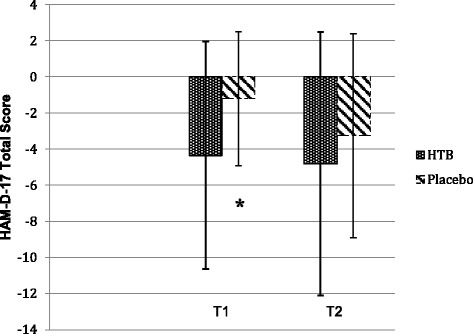
Change scores in the 17-Item Hamilton Scale for Depression (HAM-D-17) from baseline (T0) in the hyperthermic bath group compared to the placebo group (intention-to-treat population;^a^ *N* = 36). T1 indicates after 2 weeks of treatment (4 hyperthermic baths (HTB) vs sham intervention with green light (placebo); T2, 4 weeks after discontinuation of treatment (follow-up).^a^The intention-to-treat analysis used last-observation-carried-forward.Error bars indicate standard deviation.* *P* = 0.037

The ITT analysis showed a significant (*P* = .037) difference in the change in HAM-D_total score_ with 3.14 points after 4 interventions in favour of the HTB group compared to the placebo group (see Fig. 2). This result was confirmed by the PP analysis (*P* = .031) and the subscales HAM-D_insomnia_ (*P* = .048) and HAM-D_mood_ (*P* = .045) showed a significant improvement compared to the placebo group (Additional file 2: Table S1). Posthoc subgroup-analyses according to HAM-D scores quartiles (median) revealed the greatest treatment effect in quartiles 3 and 4. We found a difference of 7.88 (7.5) for HAM-D >22 at baseline in the HTB group (*n* = 8) and of 1.71 (1.7) in the placebo group (*n* = 7) (*d* = 1.10; 95% CI, 0.02 to 2.19) and a difference of 12.5 (8.2) for HAM-D >26 at baseline in the HTB group (*n* = 4) and of 1.66 (2.3) in the placebo group (*n* = 3); (*d* = 1.67; 95% CI, -0.07 to 3.40).

### Secondary outcomes

The positive effect of HTB treatment on depression remained stable until 4 weeks after the intervention without, however, reaching statistical significance (Table 3). For results of the PP analysis, see Additional file 2: Table S2 and Figures S1, S2.

**Table 3:** 17-Item Hamilton Scale for Depression: Differences between baseline and T1 (after 4 interventions) and T2 (4 weeks after treatment) in the hyperthermic bath group compared to the placebo group (intention-to-treat population;^a^ *N* = 36)

		T1				T2		
	HTB Group (*n* = 17)	Placebo Group (*n* = 19)	*P* ^§^	Cohen d^c^	HTB Group (*n* = *17*)	Placebo Group (*n* = 19)	*P* ^*§*^	Cohen d^c^
^1^HAM-D_total score_^b^	4.35 (6.3)	1.21 (3.7)	**0.037** ^*^	0.62 [-0.05-1.29]	4.82 (7.3)	3.26 (5.7)	0.238	0.24 [-0.42-0.90]
^2^HAM-D_insomnia_^b^	0.94 (1.8)	0.16 (1.0)	0.053	0.55 [-0.11-1.22]	0.88 (1.7)	0.58 (1.8)	0.306	0.17 [-0.49-0.83]
^2^HAM-D_mood_^b^	2.35 (3.2)	0.84 (2.2)	0.054	0.55 [-0.12-1.22]	2.82 (3.8)	1.74 (2.5)	0.156	0.33 [-0.33-0.99]
^2^HAM-D_somatic_^b^	0.59 (1.0)	0.21 (1.4)	0.182	0.31 [-0.35-0.97]	0.71 (1.4)	0.58 (1.4)	0.390	0.09 [-0.56-0.75]

*Abbreviations: HTB* hyperthermic baths, *HAM-D* Hamilton Scale for Depression

^a^The intention-to-treat analysis used last-observation-carried-forward

^b^Indicates differences between HTB and placebo in the change from T0 to T1/T2 to each patient’s own end point for the change in depression rating

^c^Computed as the difference between the means, M2 – M1, divided by the pooled standard deviation, sigma (σ_pooled_) of both groups. [95% confidence interval]

Data are shown as mean and standard deviation (SD)

^§^1-tailed *t* test

^1^Primary outcome

^2^Secondary outcome

*P < .05

#### Heart rate variability

Throughout the study, the data analysis (PP) did not reveal significant differences in HRV parameters between the HTB and the placebo group. Interestingly, the improvement of sleep quality (HAM-D_insomnia_) within 3 days of HTB treatment significantly correlated with an increase of nocturnal pulse rate (pearson -.63; *P*_2-tailed_ = .020) and overnight drop of the parameters LF (pearson .65; *P*_2-tailed_ = .016) and total variability of HRV (SDNN; pearson .62; *P*_2-tailed_ = .024). There was no correlation of these parameters in the sham condition.

#### Global judgement of efficacy and tolerability

The global judgement by the participants showed no significant difference between the groups, with good to moderate efficacy (HTB 2.6 (0.9); placebo 2.8 (1.0); *P*_2-tailed_ = .62) and tolerability (HTB 2.2 (1.1); placebo 1.6 (0.9); *P*_2-tailed_ = .17) after 4 interventions and good to moderate efficacy 4 weeks after intervention (HTB 2.8 (1.15); placebo 2.7 (1.10); *P*_2-tailed_ = .84).

#### Adverse events

AE were reported by 21 participants, of which 12 (86%) were assigned to the HTB group and 9 (56%) to the placebo group (Additional file 2: Table S3). No serious AE were reported by either group. There was no significant difference between the groups (*P*_2-tailed_ = .118). Typical AE in the HTB group were discomfort during the baths such as dizziness, tachycardia, tingling in the extremities and strong perspiration and thus mainly attributable to the cardiovascular system. Additionally, patients reported minor headache and nausea or a feeling of oppression, poorer sleep and increased sweating on the following days. Typical AE in the sham condition were afterimages lasting a few seconds to minutes after application, head pressure (headache) and worse sleep quality during the following night. AE resulted in 3 dropouts (DO) in the HTB group (DO_1_ weight loss, headache, exertion the day after treatment; DO_2_ anxiety at night; DO_3_ headache) and in 2 dropouts in the placebo group (DO_1_ feeling of tension; DO_2_ feeling of aggression). One dropout in the placebo group was due to lack of time. Compliance was good with a medium number of treatments of 7.3 (1.4) in the HTB group and of 7.8 (0.5) in the placebo group.

## Discussion

The main result of this pilot study was a moderate but significant improvement of 3.1 points in HAM-D_total score_ after 4 HTB treatments compared with a sham condition. The threshold for clinical significance, as established by the National Institute for Clinical Excellence (NICE), was reached with a treatment-placebo difference of 3 points on the HAM-D [33]. Cohen *d* was .62 (95% CI, -0.05 to 1.29). This is larger than the effect size of antidepressant medication in a patient-level meta-analysis with *d* = .37 [34].

In clinical trials with antidepressants, an effect size of 0.40 or higher is considered a clinically significant response criterion [35]. The effect appears even stronger if we take into account that the effect sizes in published trials of antidepressant medication are 32% higher than in unpublished trials [36]. As in pharmacological studies, the magnitude of the difference in HAM-D scores between the HTB and the placebo group increases with increasing baseline depression severity (HAM-D >22, *d* = 1.10; 95% CI, 0.02 to 2.19; HAM-D >26, *d* = 1.67; 95% CI, -0.07 to 3.40) [34]. Surprisingly, compared with the sham intervention, this did not result in a better global judgement of efficacy.

Nevertheless, these results should be interpreted with caution. First, this was a pilot study with a small sample size. Second, an improvement in HAM-D_total score_ does not necessarily indicate antidepressant action [37, 38]. On the other hand, our symptom-specific subscales show a statistically and clinically significant improvement in the dimensions “mood” and “insomnia”, at least in the PP analysis. It is a well-known fact that the HAM-D_total score_ has it pitfalls, however, for better comparability with other studies, we did not use the GRID-HAM-D e.g., with better reliability and validity [39].

Regarding feasibility we did not achieve the calculated number of 44 participants in the foreseen recruiting period, the application of the hyperthermic baths was well tolerated, we saw some minor but no severe AE, dropout rate was 18% in the HTB group and 16% in the placebo group.

The mechanism of action of HTB treatment is still unknown, but major hypotheses of WBH involve resynchronization of circadian rhythms and/or restoration of temperature physiology, resulting e.g. in better sleep [15, 16, 18]. Hence, our results are compatible with the theoretical model. We assume the difference in HAM-D scores was mainly due to an improvement in sleep quality (HAM-D_insomnia_). This is in accordance with studies on sleep disorders in non-depressed patients [19–25].

In our study, we could not see a significant effect of HTB on HRV. This may have been due to the small sample size and the high variability of HRV parameters [40]. Different effects of HTB on HRV according to age [41] may also have influenced our results, however, the sample size was too small to perform subgroup analyses.

These findings suggest that HTB treatment improves sleep quality, mediated through a 2.4 °C increase in core body temperature. Minor and medium adverse effects were encountered these mainly being discomfort and orthostatic problems arising during or directly after the baths but also disturbed sleep in some patients the night after the intervention. Besides these transient effects, HTB treatment was well tolerated.

Our results are in line with a previously published controlled study. After 2 weeks, a single session of WBH using a radiant system, with a maximum core body temperature of 38.9 °C, resulted in a decrease in HAM-D of 6 points, compared to a sham intervention [42]. This might indicate that different hyperthermia applications, fewer hyperthermic sessions, as well as lower core body or slightly higher skin temperatures give better results.

### Strengths and limitations

The strengths of our study are the randomized, placebo-controlled design, the use of standardized baths, the good control of temperature, the clinically relevant increase in core body temperature, duration of hyperthermia of about 60 min and the relatively low number of dropouts.

Several limitations should be discussed. First, because of the small sample size, the study had limited power to detect clinically significant differences between the treatment conditions, especially in subgroup analyses. Second, the absence of blinding of treatment conditions, which is inherent and inevitable, and of outcome measures (only data management and statistical analyses were performed blinded). Third, the monitoring of depressive symptoms was limited to T1 and T2.

### Generalizability

Although external validity may be restricted due to the population selected to participate in clinical studies, the population studied here can be regarded as representative of routine clinical practice, including patients with and without antidepressant medication [43]. Contraindications to HTB are still not well defined. Severe concomitant diseases, esp. cardiovascular, should be omitted especially in the elderly.

There is no hint that HTB treatments are less effective in combination with other pharmacological or non-pharmacological treatments, because we did not see a difference in outcome according to subgroups with or without antidepressant medication. There is also no theoretical reason that would compromise a combination with other non-pharmacological treatments.

## Conclusions

In conclusion, this pilot study demonstrates effects of HTB on depressive symptoms and sleep quality in depressed patients, especially in severely depressed patients, with fast onset of treatment success after 4 treatments in 2 weeks, without severe AE and with relatively good acceptance and tolerability. With HTB treatment, the core body temperature can be raised rapidly and with clinically relevant effect (2.4 °C in 20 min).

Further evaluation in rigorously designed clinical studies will be necessary to validate the impact of HTB treatment on depressive disorders. Studies should preferably explore mediators and moderators of response.

## Additional files

Additional file 1: Study protocol. (PDF 40 kb)

Additional file 2: PP analysis. **Table S1.** 17-Item Hamilton Scale for Depression: Differences between Baseline and T1 (after 4 Interventions) in the Hyperthermic Bath Group compared to the Placebo Group, *N* = 30). **Table S2.** 17-Item Hamilton Scale for Depression: Differences between Baseline and T2 (4 weeks after treatment) in the Hyperthermic Bath Group compared to the Placebo Group, *N* = 28). **Table S3.** Summary of Adverse Events. **Figure S1.** Effects of Hyperthermic Baths (HTB) and Green Light (Placebo) in Depressed Patients. **Figure S2.** Effects of Hyperthermic Baths (HTB) and Green Light (Placebo) in Depressed Patients. 17-Hamilton Scale for Depression Subscales. (DOCX 114 kb)

## Abbreviations

CVD: Cardiovascular disease
HAM-D: 17-item Hamilton Scale for Depression
HRV: Heart rate variability
HTB: Hyperthermic baths
ITT: Intention-to-treat
PP: Per-protocol
SD: Standard deviation
WBH: Whole body hyperthermia
