# Supplementary material for: Effects of hyperthermic baths on depression, sleep and heart rate variability in patients with depressive disorder: a randomized clinical pilot trial
Source: BMC Complement Altern Med. 2017 Mar 28;17:172. doi: 10.1186/s12906-017-1676-5 (PMC5371197; doi:10.1186/s12906-017-1676-5)
Supplement: Supplementary file 1 — Study protocol. (PDF 40 kb) [file 12906_2017_1676_MOESM1_ESM.pdf]

Deutsches Register Klinischer Studien
German Clinical Trials Register

PLEASE NOTE: *This trial has been registered retrospectively.*

Trial Description

Title

Hyperthermic baths in patients with depression – effects on symptoms and heart rate variability. A randomized controlled pilot study

Trial Acronym

Heat: Hyperthermia - an effective antidepressive therapy

URL of the trial

[---]*

Brief Summary in Lay Language

The biological clock which regulates biological processes in 24-hour rhythms (circadian rhythm), i.e. sleep-wake rhythm is often severely disturbed in depressive patients. Furthermore the capability of the organism to adjust the heart beat is often disturbed (heart rate variability; HRV). Hyperthermic baths increase body temperature, accentuate the circadian temperature rhythm and result, from clinical experience and small observational studies, in relevant improvement of depressive symptoms and heart rate variability when applied serially. The hypothesis of our study is, that serial hyperthermic baths improve symptoms and HRV of patients with depression. Because the effect size is, up to now, not known, this pilot study is performed which will, if the positive effects are confirmed, allow to plan a larger confirmatory study.

Brief Summary in Scientific Language

Hyperthermic baths in patients with depression – effects on symptoms and heart rate variability. A randomized controlled pilot study

Organizational Data

- DRKS-ID: **DRKS00004803**
- Date of Registration in DRKS: **2016/02/02**
- Date of Registration in Partner Registry or other Primary Registry: **[---]***
- Investigator Sponsored/Initiated Trial (IST/IIT): **yes**
- Ethics Approval/Approval of the Ethics Committee: **Approved**
- (leading) Ethics Committee Nr.: **96/13 , Ethik-Kommission der Albert-Ludwigs-Universität Freiburg**

Secondary IDs

- Other Secondary-ID: **[---]***

Health condition or Problem studied

- ICD10: **F32 - Depressive episode**
- ICD10: **F33 - Recurrent depressive disorder**

Interventions/Observational Groups

- Arm 1: **Treatment group: Hyperthermic baths.
The hyperthermic baths were applied as head-out-of-water-immersion in a 40°C pool in a spa center near Freiburg, Germany, where 5 patients at a time could sit in.
The baths had a duration until the participants noticed discomfort, with a target of 30 minutes.
Directly after the bath, the participants were accompanied to a nearby resting room, where they were layed down on a resting lounger and wrapped in warm blankets with 2 hot water bottles for at least another 30 minutes to keep the body temperature elevated.
In total participants receive 8 baths, with 2 baths per week. Follow up takes place four weeks after the last intervention.**

- Arm 2: <style pdfFontName='helvetica-bold' isBold='true'>Control group: sham intervention with green light.
Participants received a therapy with green light (<400 Lux) by an LED in a separate room in the Department of Environmental Health Sciences for 40 minutes in a sitting position.
Therapeutic effects can be expected by therapies with 10 000 Lux / 30 minutes per day.
</style>

Characteristics

- Study Type: **Interventional**
- Study Type Non-Interventional: **[---]***
- Allocation: **Randomized controlled trial**
- Blinding: **[---]***
- Who is blinded: **data analyst**
- Control: **Placebo**
- Purpose: **Treatment**
- Assignment: **Parallel**
- Phase: **N/A**
- Off-label use (Zulassungsüberschreitende Anwendung eines Arzneimittels): **No**

Primary Outcome

Hamilton Rating Scale for Depression-17 (total score): Difference between baseline and T1 (after 4 interventions) in the intervention group compared to the control group.

Secondary Outcome

• Hamilton Rating Scale for Depression-17 (total score): Difference between groups 4 weeks after end of treatment (follow up).

• Hamilton Rating Scale for Depression-17 sub scales: Difference between groups after 4 treatments and 4 weeks after end of treatment (follow up).

• Heart Rate Variability (parameter): Total Heart Rate Variability, Low Frequency, High Frequency) and heart rate during sleep (21:00 - 04:00 h) after 4 treatments and 4 weeks after end of treatment (follow up) compared to the control group and correlation with Hamilton Rating Scale for Depression-17 (total score) and subscales.

• Adverse effects.
• Global judgement of efficacy and tolerability on a 5-item scale by the participants.
•Core body temperature measured by an ear thermometer before and after the bath and after the resting time.

Countries of recruitment

- DE **Germany**

Locations of Recruitment

- University Medical Center **Uni-Zentrum Naturheilkunde, Freiburg im Breisgau**

Recruitment

- Planned/Actual: **Actual**
- (Anticipated or Actual) Date of First Enrollment: **2013/05/01**
- Target Sample Size: **44**
- Monocenter/Multicenter trial: **Monocenter trial**
- National/International: **National**

Inclusion Criteria

-

Gender: **Both, male and female**

- Minimum Age: **18 Years**
- Maximum Age: **65 Years**

Additional Inclusion Criteria

**•Age 18 – 65 years
•male or female
•Depression (ICD F32/33) lasting longer than 4 weeks confirmed by a physician or psychotherapist
•Hamilton Depression Scale Total Score ≥18 and a score ≥2 Item "depressive mood" at Screening and Baseline.
•No change of antidepressant medication during the last 4 weeks and the trial foreseen**

Exclusion criteria

pregnancy, lactation, current and serious suicidal or homicidal risk (according to investigator's judgment), serious concomitant disease (e.g. heart failure, renal failure, lung disease, open wounds), history of seizure disorder, organic mental disorders, alcohol or substance use within the last 6 months prior to the screen visit, schizophrenia, delusional disorder, psychotic disorders, bipolar disorder, antisocial personality disorder, heat urticaria, change of the antidepressive treatment within 4 weeks prior to the screen visit, treatment with beta-blocker or steroids and patients who don`t like hot baths.

Addresses

- **Primary Sponsor**

**Uni-Zentrum Naturheilkunde
Mr. PD Dr. Roman Huber
Breisacher Str. 115b
79106 Freiburg
Germany**

Telephone: **0761 270 82010**
Fax: **[---]***
E-mail: **roman.huber at uniklinik-freiburg.de**
URL: **[---]***

- **Contact for Scientific Queries**

Uni-Zentrum für Naturheilkunde / Department of Environmental Health Sciences Universitätsklinikum Freiburg

Mr. PD Dr. med. Roman Huber
Breisacher Str. 115b
79106 Freiburg
Germany

Telephone: **0761/270-82010**
Fax: **0761/270-83230**
E-mail: **roman.huber at uniklinik-freiburg.de**
URL: **http://www.uniklinik-freiburg.de/iuk/live/unizentrumnaturheilkunde.html**

- **Contact for Public Queries**

Uni-Zentrum für Naturheilkunde / Department of Environmental Health Sciences Universitätsklinikum Freiburg

Ms. cand. med. Sonja Kaifel
Breisacher Str. 115b
79106 Freiburg
Germany

Telephone: **0761 270-83120**
Fax: **[---]***
E-mail: **sonja.kaifel at uniklinik-freiburg.de**
URL: **[---]***

Sources of Monetary or Material Support

- **Institutional budget, no external funding (budget of sponsor/PI)**

Uni-Zentrum Naturheilkunde / Institut für Umweltmedizin und Krankenhaushygiene /Universitätsklinikum Freiburg
Mr. PD Dr. med. Roman Huber
Breisacher Str. 115b
79106 Freiburg
Germany

Telephone: **0761/270-82010**
Fax: **0761/270-83230**
E-mail: **roman.huber at uniklinik-freiburg.de**
URL: **[---]***

Status

- Recruitment Status: **Recruiting complete, follow-up complete**
- Study Closing (LPLV): **2013/12/12**

Trial Publications, Results and other documents

** This entry means the parameter is not applicable or has not been set.*
