# Supplementary material for: Effects of hyperthermic baths on depression, sleep and heart rate variability in patients with depressive disorder: a randomized clinical pilot trial
Source: BMC Complement Altern Med. 2017 Mar 28;17:172. doi: 10.1186/s12906-017-1676-5 (PMC5371197; doi:10.1186/s12906-017-1676-5)

**Additional file 1**

- **Table S1. 17-Item Hamilton Scale for Depression: Differences between Baseline and T1 (after 4 Interventions) in the Hyperthermic Bath Group compared to the Placebo Group, N = 30).**
- **Table S2. 17-Item Hamilton Scale for Depression: Differences between Baseline and T2 (4 weeks after treatment) in the Hyperthermic Bath Group compared to the Placebo Group, N = 28).**
- **Table S3. Summary of Adverse Events**
- **Figure S1. Effects of Hyperthermic Baths (HTB) and Green Light (Placebo) in Depressed Patients.**
- **Figure S2. Effects of Hyperthermic Baths (HTB) and Green Light (Placebo) in Depressed Patients. 17-Hamilton Scale for Depression Subscales.**

**Table S1. 17-Item Hamilton Scale for Depression: Differences between Baseline and T1 (after 4 Interventions) in the Hyperthermic Bath Group compared to the Placebo Group, N = 30).**

|  |  | | **HTB**  **Group**  **N = 14** | **Placebo**  **Group**  **N = 16** | *P*^§^ | **Cohen**  **d^b^** |
| --- | --- | --- | --- | --- | --- | --- |
|  | | ^1^HAM-D_total score_^a^ | 5.29 ± 6.62 | 1.44 ± 4.02 | **0.031*** | 0.72  [-0.03-1.46] |
|  | ^2^HAM-D_insomnia_^a^ | | 1.14 ± 1.88 | 0.19 ± 1.11 | **0.048*** | 0.63  [-0.11-1.36] |
|  | ^2^HAM-D_mood_^a^ | | 2.86 ± 3.37 | 1.00 ± 2.37 | **0.045*** | 0.65  [-0.09-1.38] |
|  | ^2^HAM-D_somatic_^a^ | | 0.71 ± 1.07 | 0.25 ± 1.53 | 0.175 | 0.34  [-0.38-1.07] |

Abbreviations: HTB, hyperthermic baths; HAM-D, Hamilton Scale for Depression.

^a^Indicates differences between HTB and placebo in the change from T0 to T1 to each patient’s own end point for the change in depression rating.

^b^Computed as the difference between the means, M2 − M1, divided by the pooled standard deviation, sigma (σ_pooled_) of both groups. Data are shown as mean and standard deviation (SD).

^§^1-tailed *t* test.

^1^Primary outcome.

^2^Secondary outcome.

**P* < .05.

**Table S2. 17-Item Hamilton Scale for Depression: Differences between Baseline and T2 (4 weeks after treatment) in the Hyperthermic Bath Group compared to the Placebo Group, N = 28*).**

|  | |  | | | **HTB**  **Group**  **N = 13** | | **Placebo**  **Group**  **N = 15** | | *P^§^* | | **Cohen**  **d^b^** |  |
| --- | --- | --- | --- | --- | --- | --- | --- | --- | --- | --- | --- | --- |
|  | | | ^1^HAM-D_total score_^a^ | | 6.38 ± 7.74 | | 4.13 ± 6,105 | | 0.199 | | 0.33  [-0.42-1.07] |  |
|  | | ^2^HAM-D_insomnia_^a^ | | | 1.15 ± 1.91 | | 0.80 ± 1.97 | | 0.317 | | 0.18  [-0.56-0.92] |  |
|  | | ^2^HAM-D_mood_^a^ | | | 3.62 ± 4.05 | | 2.00 ± 2.65 | | 0.109 | | 0.48  [-0.27-1.23] |  |
|  | | ^2^HAM-D_somatic_^a^ | | | 0.92 ± 1.50 | | 0.80 ± 1.42 | | 0.413 | | 0.08  [-0.66-0.83] | |

Abbreviations: HTB, hyperthermic baths; HAM-D, Hamilton Scale for Depression.

^a^Indicates differences between HTB and placebo in the change from T0 to T2 to each patient’s own end point for the change in depression rating.

^b^Computed as the difference between the means, M1 − M2, divided by the pooled standard deviation, sigma (σ_pooled_) of both groups.

Data are shown as mean ± standard deviation (SD).

^§^1-tailed *t* test.

^1^Primary outcome.

^2^Secondary outcome.

* missing data from 2 participants

**Table S3. Summary of Adverse Events**

|  | **No. (%)** | |
| --- | --- | --- |
| **Adverse Events** | **HTB Group**  **(n = 14)** | **Placebo Group**  **(n = 16)** |
| No adverse events | 2 (14) | 7 (44) |
| Minor adverse events | 10 (71) | 8 (50) |
| Moderate adverse events | 2 (14) | 1 (6) |
| Severe adverse events | 0 (0) | 0 (0) |
| Adverse events total | 12 (86) | 9 (56) |
|  |  |  |

**Figure S1. Effects of Hyperthermic Baths (HTB) and Green Light (Placebo) in Depressed Patients.**


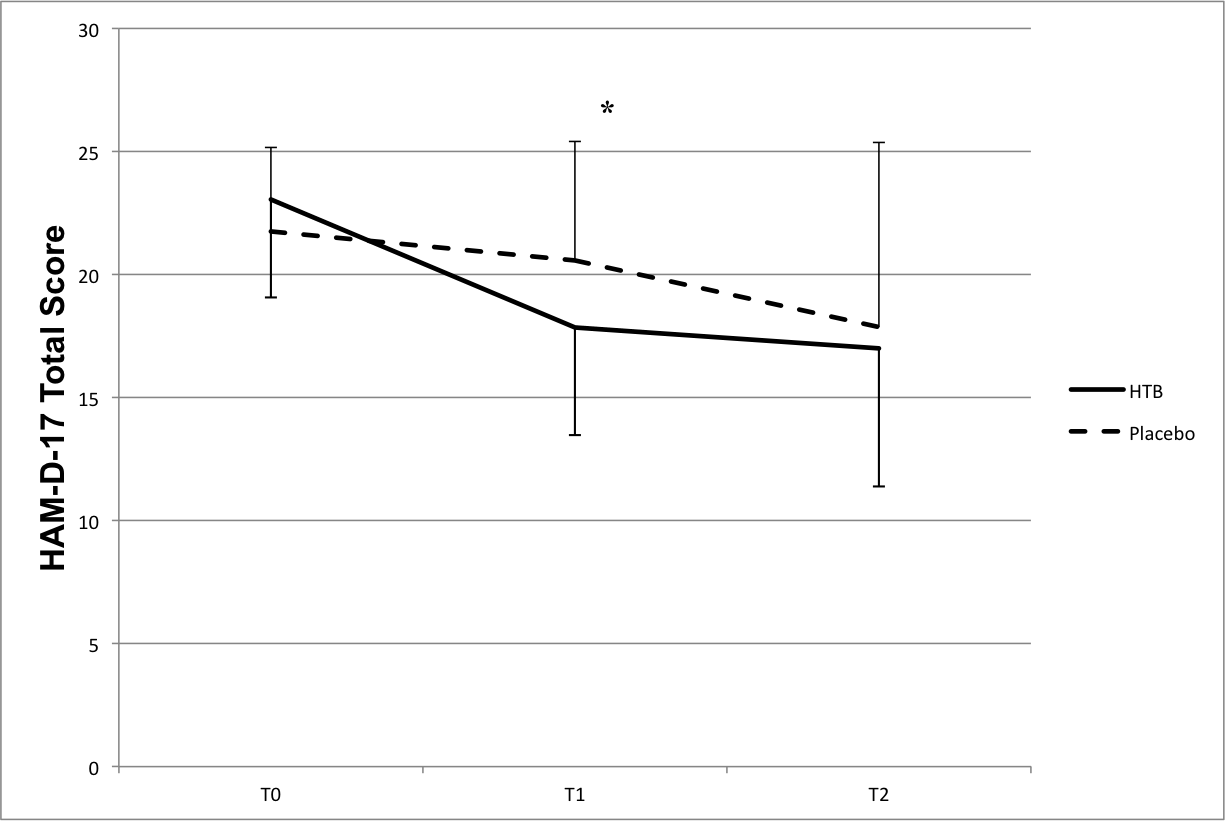


Data are depicted as means; error bars show the standard deviation.

Measures include the 17-Hamilton Scale for Depression total scores.

T0 N=36 (n=17 HTB group, n=19 placebo group).

T1 indicates after 2 weeks of treatment (4 HTB), N=30 (n=14 HTB group, n=16 placebo group).

T2 indicates 4 weeks after discontinuation of treatment, N=28 (n=13 HTB group, n=15 placebo group).

****P* = .031.**

**Figure S2. Effects of Hyperthermic Baths (HTB) and Green Light (Placebo)**

**in Depressed Patients. 17-Hamilton Scale for Depression Subscales.**

T0 T1 T2

*****

****P* = .045***

****P* = .048***

*****

T0 T1 T2


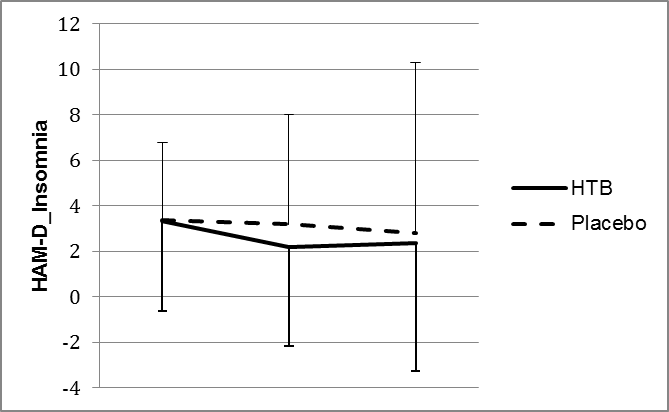


*****

Data are depicted as means; error bars show the standard deviation.

T0 N=36 (n=17 HTB group, n=19 placebo group).

T1 indicates after 2 weeks of treatment (4 HTB), N=30 (n=14 HTB group, n=16 placebo group).

T2 indicates 4 weeks after discontinuation of treatment, N=28 (n=13 HTB group, n=15 placebo group).

T0 T1 T2


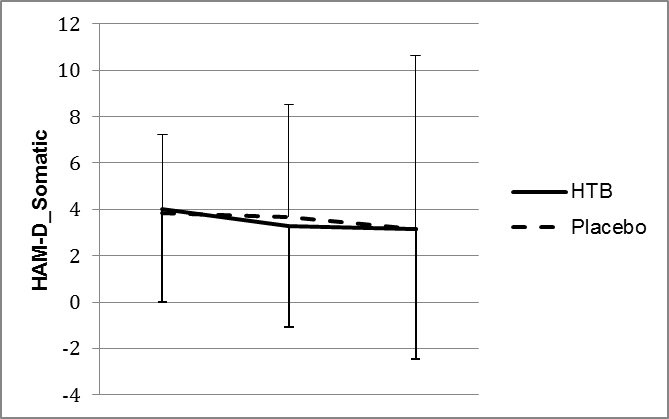

Supplement: Supplementary file 2 — PP analysis. Table S1. 17-Item Hamilton Scale for Depression: Differences between Baseline and T1 (after 4 Interventions) in the Hyperthermic Bath Group compared to the Placebo Group, N = 30). Table S2. 17-Item Hamilton Scale for Depression: Differences between Baseline and T2 (4 weeks after treatment) in the Hyperthermic Bath Group compared to the Placebo Group, N = 28). Table S3. Summary of Adverse Events. Figure S1. Effects of Hyperthermic Baths (HTB) and Green Light (Placebo) in Depressed Patients. Figure S2. Effects of Hyperthermic Baths (HTB) and Green Light (Placebo) in Depressed Patients. 17-Hamilton Scale for Depression Subscales. (DOCX 114 kb) [file 12906_2017_1676_MOESM2_ESM.docx]
